# Associations of a nursing care bundle with clinical outcomes in patients with severe burns and inhalation injury: a retrospective cohort study

**DOI:** 10.3389/fmed.2026.1809662

**Published:** 2026-05-07

**Authors:** Jinmei Cheng, Zhiqing Sun, Yumeng Wang, Mengyao Chu, Jia Miao

**Affiliations:** Department of Burn Plastic Surgery and Wound Repair, Huai’an First People’s Hospital, Affiliated to Nanjing Medical University, Huai’an, Jiangsu, China

**Keywords:** burns, catheter-related bloodstream infection, diabetic foot, inhalation injury, nursing care bundle, ventilator-associated pneumonia

## Abstract

**Aim:**

To evaluate the associations of a comprehensive nursing care bundle on clinical outcomes in patients with severe burns and inhalation injury.

**Methods:**

A retrospective cohort study was conducted on 130 patients admitted to a burn ICU from July 2022 to December 2024. Patients were divided into a Comprehensive Care Group (Group A, *n* = 65) receiving a protocol of ultrasound-guided venous catheterization through burn wounds, tracheostomy via burn wounds, systematic air-fluidized bed management, and intensified diabetic foot prevention, and a Conventional Care Group (Group B, *n* = 65).

**Results:**

Group A was associated with lower rates of catheter-related bloodstream infection (1.6% vs. 11.3%, *p* = 0.033), delayed onset of ventilator-associated pneumonia, reduced back wound deterioration (12.3% vs. 40.0%, *p* < 0.001), and zero incidence of diabetic foot in the diabetic subgroup (0% vs. 47.1%, *p* < 0.001). Overall, Group A had shorter hospital stays, lower MODS incidence (15.4% vs. 36.9%, *p* = 0.005), and reduced mortality (7.7% vs. 21.5%, *p* < 0.001).

**Conclusion:**

In patients with severe burns complicated by inhalation injury, implementation of the diversified bundled nursing strategy was associated with improved clinical outcomes and appeared to be safe in this retrospective cohort. However, because of the retrospective design and potential residual confounding, these findings should be interpreted with caution and should not be considered definitive evidence of causality.

## Introduction

Burns represent a common type of clinical trauma, and severe burns are frequently complicated by inhalation injury, constituting a life-threatening, complex critical condition (1). Such patients face not only extensive destruction of body surface tissues but also direct damage to the respiratory tract from thermal energy, smoke, and toxic chemicals. This leads to airway edema, obstruction, pulmonary parenchymal injury, and exacerbation of systemic inflammatory response, increasing the difficulty of clinical management and mortality (2). Although traditional rescue systems are continuously advancing, there remains a lack of sufficient evidence-based support for systematic nursing protocols targeting this specific population (3). Particularly when multiple high-difficulty, high-risk nursing needs converge, how to make decisions and optimize the nursing pathway becomes a critical factor influencing patient prognosis (4).

In clinical practice, the nursing of patients with severe burns combined with inhalation injury faces a series of unique and interrelated challenges. The primary challenge is the establishment of safe and effective life-sustaining access. When a patient has extensive body surface burns with extremely limited intact skin available for puncture, conventional venous catheterization and tracheotomy routes may be unfeasible or carry increased risks (5). In recent years, sporadic reports have explored the possibility of performing invasive procedures through burn wounds (6). However, widespread concerns persist regarding their safety, especially the risk of subsequent catheter-related infections or the spread of tracheostomy site infections, often leading to clinical dilemmas in decision-making (7). Secondly, due to the patient’s inability to change position independently, effectively managing the extensive wounds and preventing progressive deepening of wounds at pressure points as well as pressure ulcer formation presents another persistent challenge (8). The application of devices such as air-fluidized beds and turning frames provides physical solutions, but a clear consensus on the optimal usage patterns, timing, and their synergistic effects with other nursing measures has not yet been established (9). More complex is the fact that severe burns themselves trigger an intense stress response, leading to insulin resistance and severe blood glucose fluctuations (10). This dramatically increases the probability of severe complications such as diabetic foot occurring during the acute phase and rehabilitation period in patients with pre-existing diabetes or at risk for diabetes (11). However, during the acute phase where life-saving is the priority, preventive nursing care for such long-term complications is often neglected or delayed (12).

Therefore, there is an urgent clinical need for an integrated and highly operable nursing strategy capable of systematically addressing the aforementioned multi-dimensional challenges simultaneously, rather than dealing with individual problems in isolation (13). This strategy should be prospective and coordinated, capable of transforming seemingly contradictory operations (such as establishing access through wounds) into safe choices based on precise assessment and rigorous monitoring (14). It should also organically link nursing measures from different aspects (such as posture management and metabolic regulation) to create a synergistic, additive effect (15).

This study aims, through a retrospective cohort analysis, to explore the application effect of a diversified protocol integrating four key nursing strategies in patients with severe burns and inhalation injury. The core of this protocol includes: prudently performed ultrasound-guided central venous catheterization through the burn wound, tracheotomy through the burn wound based on a structured assessment of local wound status, including burn depth, the presence of necrotic tissue or obvious infection, local contamination/exudation, tissue stability, and whether the underlying target vessel or tracheotomy pathway could be safely identified, with ultrasound guidance used when applicable. We hypothesize that compared to conventional nursing following traditional pathways, this actively integrated, individually implemented care bundle strategy might be associated with reduced incidence of complications such as catheter-related infections, ventilator-associated pneumonia, progressive wound deterioration, and diabetic foot, ultimately improving patients’ organ function status and survival outcomes. The results of this study are expected to provide valuable clinical evidence for constructing more refined and systematic practical guidelines for critical burn nursing.

## Materials and methods

### Study subjects

This study is a single-center retrospective cohort study. The study subjects were derived from all patients admitted to the Burn Intensive Care Unit (BICU) of our hospital between July 1, 2022, and December 31, 2024, who met the pre-defined criteria. All diagnostic, therapeutic, and nursing procedures followed established departmental protocols. This study solely conducted subsequent analysis of the generated clinical data without intervening in the treatment process. The requirement for informed consent was waived after review and approval by the hospital ethics committee.

The inclusion criteria for patients were strictly set as follows: (1) Age not less than 18 years; (2) Total body surface area burned (TBSA) greater than or equal to 30%, to ensure the inclusion of a moderate-to-severe burn patient population; (3) A definitive diagnosis of inhalation injury confirmed by clinical assessment combined with fiberoptic bronchoscopy; inhalation injury severity was subsequently categorized as mild, moderate, or severe for baseline description and analysis; (4) An anticipated survival time exceeding 72 h, to exclude cases of very early mortality that could interfere with the evaluation of nursing measure effectiveness. Exclusion criteria included: (1) Presence of definitive septic shock or multiple organ dysfunction syndrome (MODS) at admission; (2) Comorbidity with uncontrolled malignant tumor or severe congenital immunodeficiency disease; (3) Clinical medical records lacking key information, preventing effective data extraction.

### Standard burn care at our center

All enrolled patients received standard burn critical care according to our institutional practice. Burn size was estimated using TBSA assessment, and burn depth was evaluated by experienced burn surgeons based on serial clinical examination. Mechanical ventilation was provided when indicated for inhalation injury, airway protection, respiratory failure, or perioperative management, with adjustment according to blood gas analysis and clinical respiratory parameters. Nutritional management followed an enteral-first principle, with parenteral nutrition used when enteral feeding was not feasible or insufficient. Sedation and analgesia were individualized according to pain severity, agitation, ventilator synchrony, and procedural requirements. Wound care included regular wound assessment, dressing changes, debridement or escharectomy when indicated, infection control, and staged grafting as needed. Following improvement in critical illness, patients were transferred from the BICU to the burn ward or another appropriate inpatient unit for continued treatment and rehabilitation.

### Grouping basis and interventions

The final grouping of study subjects was retrospectively determined according to predefined operational criteria based on the actual nursing pathway received during hospitalization. Patients were assigned to Group A if they were managed under the integrated comprehensive care pathway, defined by the coordinated implementation of the major study interventions as a bundled strategy. Patients were assigned to Group B if they were managed under the conventional care pathway and did not receive the integrated bundled approach. For patients who received only some individual components of the study interventions, classification was based on the principal nursing strategy pattern implemented throughout hospitalization, rather than the receipt of any single component in isolation.

Group A was defined as the “Comprehensive Care Group.” Patients in this group received an integrated nursing strategy pre-defined and proactively planned by the research team. Specifically, for patients requiring central venous access, the nursing team participated in pre-procedural assessment and coordination, and qualified physicians performed ultrasound-guided catheterization. In Group A, vessels beneath eschar or deep burn wound areas were preferentially selected when the vascular structures were clearly identifiable and deemed suitable for access. For patients requiring a definitive artificial airway, tracheostomy was performed by experienced physicians/surgeons. If burn wounds were present on the neck, a trans-wound tracheostomy approach was considered after structured assessment of the local wound condition and procedural feasibility. All patients in Group A received treatment on an air-fluidized bed (e.g., Clinitron bed) during the acute phase when posterior trunk burns were present and at highest risk for pressure-related wound deterioration. While on the air-fluidized bed, patients underwent systematic positional changes according to a standardized turning schedule (every 2–4 h), performed manually or with mechanical assistance. After posterior wounds stabilized or following skin grafting, patients were transitioned to standard pressure redistribution mattresses with continued scheduled turning. For patients with a confirmed diagnosis of diabetes at admission or whose post-admission blood glucose monitoring indicated hypermetabolic risk, a standardized diabetic foot prevention protocol was immediately initiated. This protocol included daily assessment of foot skin integrity, use of offloading devices, preventive foot care, and patient/family education. In this study, these invasive procedures were not independently performed by nurses; rather, they were incorporated into the bundled nursing strategy because nursing staff played a key role in assessment, preparation, procedural assistance, maintenance, monitoring, and complication prevention within the multidisciplinary care pathway.

Group B was defined as the “Conventional Care Group.” The care received by patients in this group followed the conventional practice pathway of our center prior to this study period. Specifically, it included: Strict avoidance of puncture through any visible burn wound; central venous catheterization exclusively choosing traditional non-burn sites such as the internal jugular, subclavian, or femoral veins. Tracheotomy strictly adhered to aseptic surgical principles, performed on relatively intact skin areas after debridement or at conventional anatomical sites. Posture management primarily relied on standard air mattresses combined with manual turning based on nurses’ experience, without mandatory use of air-fluidized beds or standardized turning frequency. For diabetic or hyperglycemic patients, the nursing focus was on blood glucose monitoring and control, without systematically implementing the aforementioned specialized foot prevention protocol.

### Observation indicators and data collection

To comprehensively evaluate the effects of the two strategies, the research team constructed a multi-dimensional observation indicator system. All data were extracted independently by two uniformly trained researchers using a double-blind method through reviewing the hospital’s Electronic Medical Record (EMR) system, nursing documentation system, and some paper records. Any discrepancies were resolved by a third-party arbitrator to ensure data accuracy and consistency.

Primary indicators were patient baseline characteristics, including demographic information (age, sex), burn severity indicators (TBSA, full-thickness burn area), inhalation injury grade (categorized as mild, moderate, or severe based on bronchoscopic findings of mucosal edema, congestion, and necrosis), Acute Physiology and Chronic Health Evaluation II (APACHE II) (16) score at admission, and key comorbidities such as diabetes.

Nursing process and complication indicators were central to the evaluation. For venous access, data were recorded for each individual central venous catheterization episode, including the date of insertion, anatomical site, whether the catheter was placed through a burn wound, whether ultrasound guidance was used, the indwelling duration of that catheter, the reason for catheter removal, and the occurrence of catheter-related local infection (CRI) or catheter-related bloodstream infection (CRBSI). If a patient required more than one central venous catheter during hospitalization, each catheter episode was recorded and analyzed separately. Thus, “catheter indwelling time” in this study refers to the indwelling time of each catheter rather than the cumulative catheter days per patient. CRI was defined as localized signs of infection at the catheter insertion/exit site or along the catheter tract, including erythema, tenderness, swelling, warmth, or purulent drainage. CRBSI was defined according to CDC/NHSN criteria as a laboratory-confirmed bloodstream infection in a patient with an eligible central line in place for more than 2 consecutive calendar days on the date of event or the day before, with no evidence that the bloodstream infection was secondary to infection at another body site. Thus, bloodstream infections with a documented alternative source were not classified as catheter-related bloodstream infection in this study (17, 18). Diagnosis of the latter two strictly followed Centers for Disease Control and Prevention (CDC) criteria. For airway management, tracheotomy date, approach, airway secretion characteristic scores on postoperative day 3 and day 7 (using a validated 4-point scale where higher scores indicate thicker secretions), and the date and diagnosis of ventilator-associated pneumonia (VAP) (based on a clinical pulmonary infection score ≥6 and supported by microbiological evidence) were recorded (19). For wounds and skin, focused documentation included changes in wound depth at major pressure areas like the back and sacrococcygeal region assessed at admission and weekly, determining whether progression from deep partial-thickness to full-thickness (wound deterioration) occurred, and recording any Stage II or higher pressure injury events (according to the National Pressure Ulcer Advisory Panel staging system) (20). For diabetic foot, within the diabetic patient subgroup, documentation included whether foot ulcers of Wagner (21) grade 1 or higher occurred during hospitalization and within a 3-month follow-up period after discharge.

Final outcome indicators included: total hospital length of stay (from admission to discharge or death date), occurrence of MODS (using the Marshall scoring system (22), where a score ≥2 for any organ system indicated dysfunction), in-hospital all-cause mortality, and discharge outcome status (cured and discharged, discharged against medical advice, died). Simultaneously, data on potential confounding factors that might affect results were collected, such as timing of the first escharectomy/debridement surgery (days post-burn), cumulative days of broad-spectrum antibiotic use (e.g., carbapenems, glycopeptides), and average blood glucose level during hospitalization, for subsequent statistical adjustment.

### Statistical analysis

All collected data were entered into the professional statistical software SPSS 27.0 (IBM Corp., Armonk, NY, USA) for analysis. For continuous data, normality was first tested. Data conforming to normal distribution are described as mean ± standard deviation (x ± s), and intergroup comparisons were made using the independent samples t-test. Non-normally distributed data are described as median and interquartile range, with intergroup comparisons made using the Mann–Whitney U test. Categorical data are described as frequency and percentage, with intergroup comparisons made using the chi-square test or Fisher’s exact test. To dynamically display the risk of time-related events (e.g., CRBSI, VAP), survival curves were plotted using the Kaplan–Meier method, and intergroup comparisons were made using the Log-rank test. Cox proportional hazards regression was used to evaluate the association between group assignment and time-to-event outcomes, with hazard ratios (HRs) and 95% confidence intervals (CIs) reported. All statistical analyses employed two-sided tests, and a *p*-value less than 0.05 was considered statistically significant.

## Results

### Comparative analysis of basic characteristics of study subjects

A total of 187 patients admitted to the BICU between July 2022 and December 2024 were screened. Fifty-seven patients were excluded according to predefined criteria, including age <18 years, TBSA <30%, no inhalation injury or mild inhalation injury only, and anticipated survival ≤72 h. The remaining 130 eligible patients were retrospectively grouped according to the actual nursing interventions received into Group A (Comprehensive Care Group, *n* = 65) and Group B (Conventional Care Group, *n* = 65). A flow chart summarizing patient screening, exclusions by criterion, and final group allocation is provided as Supplementary Figure S1. The two groups demonstrated good balance in demographic and clinical baseline characteristics. The mean ages were (43.7 ± 11.1) years and (47.1 ± 14.1) years, respectively (*p* = 0.125), and the male proportions were 72.3 and 67.7%, respectively (*p* = 0.566). Core indicators reflecting burn severity, namely TBSA [(55.1 ± 16.6)% vs. (54.2 ± 17.2)%, *p* = 0.750] and full-thickness burn area [(28.6 ± 12.3)% vs. (25.7 ± 12.4)%, *p* = 0.185], showed no statistically significant differences between groups. The distribution of inhalation injury grades, admission APACHE II scores [(16.4 ± 4.9) points vs. (17.4 ± 6.5) points, *p* = 0.322], and the prevalence of comorbid diabetes (27.7% vs. 26.2%, *p* = 0.843) also showed no significant differences (all *p* > 0.05). However, Group A showed a numerically higher proportion of mild inhalation injury (30.8% vs. 15.4%) and a lower proportion of severe inhalation injury (12.3% vs. 15.4%), which should be considered when interpreting subsequent outcome differences. These data indicate that the two groups were broadly comparable in terms of disease severity and key confounding factors, but residual differences in respiratory severity cannot be fully excluded (Table 1). When comparing admission organ dysfunction using the MODS scoring system, no significant differences were observed between the two groups in any of the six organ systems or the total MODS score (all *p* > 0.05, Table 1), further supporting the baseline comparability of the study groups.

**Table 1:** Comparison of baseline characteristics between the comprehensive care group.

Characteristic	Group A (*n* = 65)	Group B (*n* = 65)	Statistical test	*p*-value
Age (years, x̄±s)	43.7 ± 11.1	47.1 ± 14.1	*t*-test	0.125
Sex (Male, *n*, %)	47 (72.3%)	44 (67.7%)	*χ*^2^ test	0.566
TBSA (%, x̄±s)	55.1 ± 16.6	54.2 ± 17.2	*t*-test	0.750
Full-Thickness Burn Area (%, x̄±s)	28.6 ± 12.3	25.7 ± 12.4	*t*-test	0.185
Inhalation Injury Grade (*n*, %)			*χ*^2^ test	0.114
Mild	20 (30.8%)	10 (15.4%)		
Moderate	37 (56.9%)	45 (69.2%)		
Severe	8 (12.3%)	10 (15.4%)		
APACHE II Score (x̄±s)	16.4 ± 4.9	17.4 ± 6.5	*t*-test	0.322
MODS Organ Scores at Admission, median (IQR)			Mann–Whitney U	
Respiratory (PaO₂/FiO_2_)	280 (240–320)	270 (230–310)		0.412
Renal (creatinine, μmol/L)	75 (60–95)	80 (62–98)		0.568
Hepatic (bilirubin, μmol/L)	12 (8–18)	13 (9–20)		0.734
Cardiovascular (PAR)	2.0 (1.5–2.5)	2.0 (1.5–3.0)		0.891
Coagulation (platelets, ×10^9^/L)	180 (150–220)	175 (140–210)		0.623
Neurological (GCS)	14 (12–15)	13 (11–15)		0.385
Total MODS Score, median (IQR)	4 (3–6)	5 (3–7)	Mann–Whitney U	0.427
Comorbid diabetes (*n*, %)	18 (27.7%)	17 (26.2%)	*χ*^2^ test	0.843

TBSA, total body surface area burned; APACHE II, Acute Physiology and Chronic Health Evaluation II.

### Associations of wound-traversing venous access with infection outcomes

**Figure 1:**
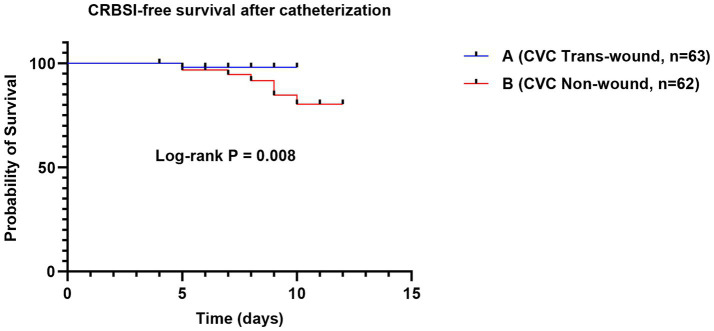
Comparison of venous access outcomes between wound-traversing and non-wound catheterization strategies. CRI: catheter-related local infection; CRBSI: catheter-related bloodstream infection; ns, not significant (*p* > 0.05). Data are presented as mean ± standard deviation; Statistical comparisons were performed using the Chi-square test or Fisher’s exact test as appropriate. ***p < 0.001; *p < 0.05.

**Figure 2:**
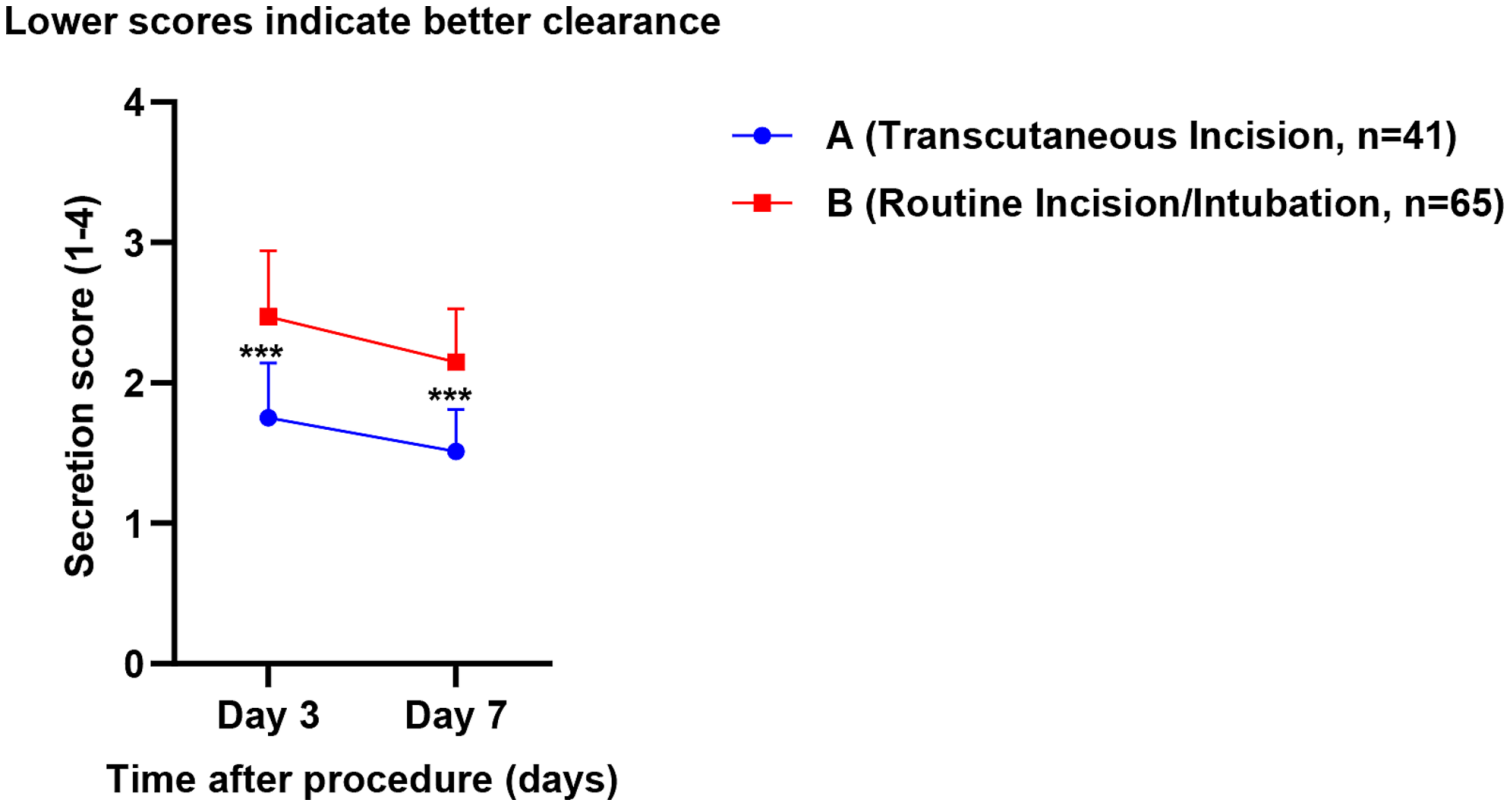
Cumulative survival curves for freedom from catheter-related bloodstream infection (CRBSI).

Among the 125 patients requiring central venous access, the two groups adopted distinctly different strategies. The majority of Group A patients (96.9%, 63/65) received ultrasound-guided catheterization through burn wounds, whereas the majority of Group B patients (95.4%, 62/65) received catheterization in non-burn areas. Analysis of catheter-level data (Figure 1) showed that the mean indwelling duration per catheter episode was shorter in the wound-traversing group than in the non-wound group [(6.70 ± 1.93) days vs. (8.29 ± 2.34) days, *p* < 0.001]. Regarding infectious complications, there was no statistical difference in the incidence of CRI between the two groups (9.5% vs. 11.3%, *p* = 0.778). However, the key CRBSI incidence was only 1.6% in the wound-traversing group, lower than the 11.3% in the non-wound group (*p* = 0.033). Kaplan–Meier survival curves (Figure 2) further dynamically confirmed that the cumulative CRBSI-free survival rate was consistently higher in Group A than in Group B (Log-rank *p* < 0.05).

### Associations of wound-traversing airway management with respiratory outcomes

Among all 130 patients, there was a fundamental difference in the strategy for establishing invasive artificial airways. All patients in both groups required invasive mechanical ventilation due to moderate or severe inhalation injury. Within the Comprehensive Care Group (Group A), 63.1% (41/65) of patients underwent tracheotomy through neck burn wounds as part of their proactive care strategy. The remaining 24 patients (36.9%) in Group A received orotracheal or nasotracheal intubation instead of tracheotomy. Reasons for not performing tracheotomy included absence of neck burn wounds (*n* = 10), unfavorable local wound conditions such as active infection or unstable eschar (*n* = 8), and clinician preference based on anticipated short duration of mechanical ventilation (*n* = 6). In the Conventional Care Group (Group B), all patients (65/65, 100%) strictly followed conventional pathways: 41 patients (63.1%) received orotracheal or nasotracheal intubation, and 24 patients (36.9%) underwent tracheotomy through intact skin at conventional anatomical sites without traversing burn wounds. Therefore, this study compared the management effects between the subgroup of Group A who underwent wound-traversing incision (*n* = 41) and the entire Group B (*n* = 65) within the framework of all patients receiving invasive airway ventilation.

**Figure 3:**
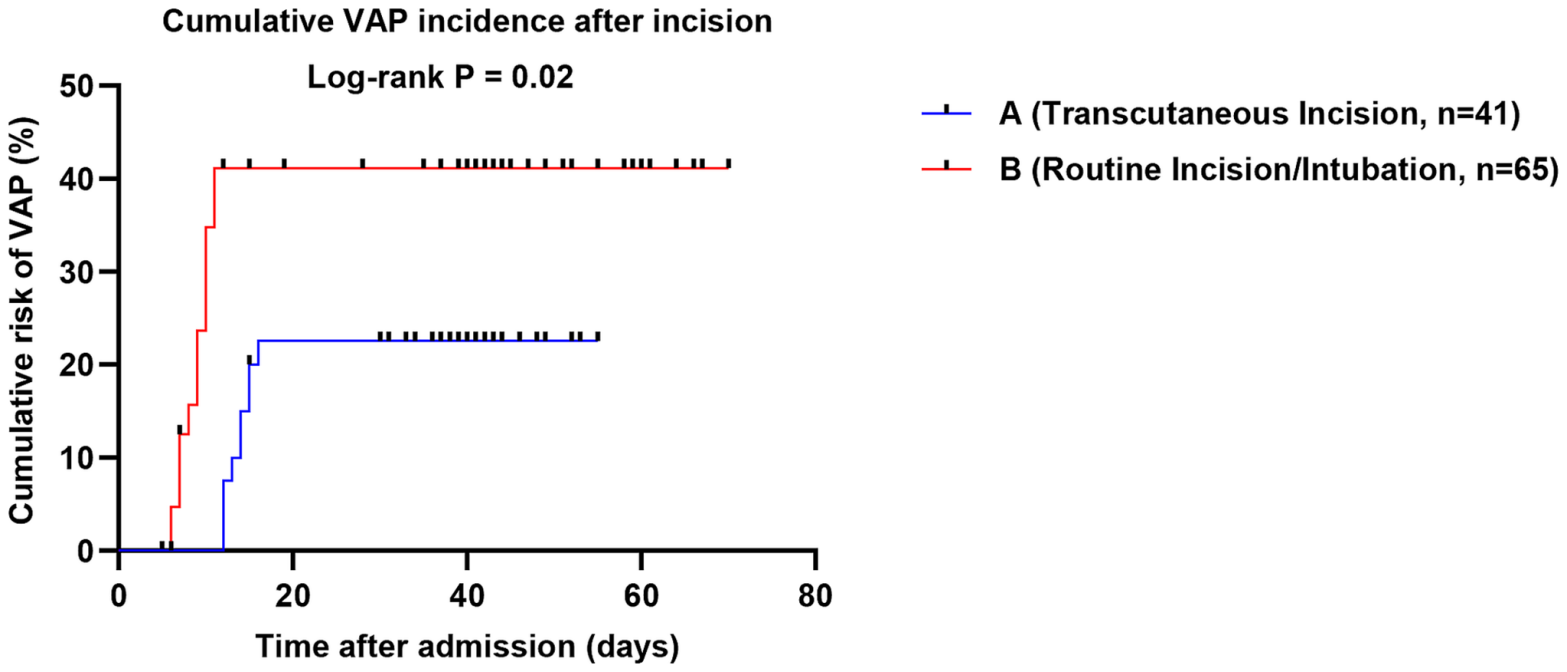
Trend of postoperative airway secretion scores in the two groups.

**Figure 4:**
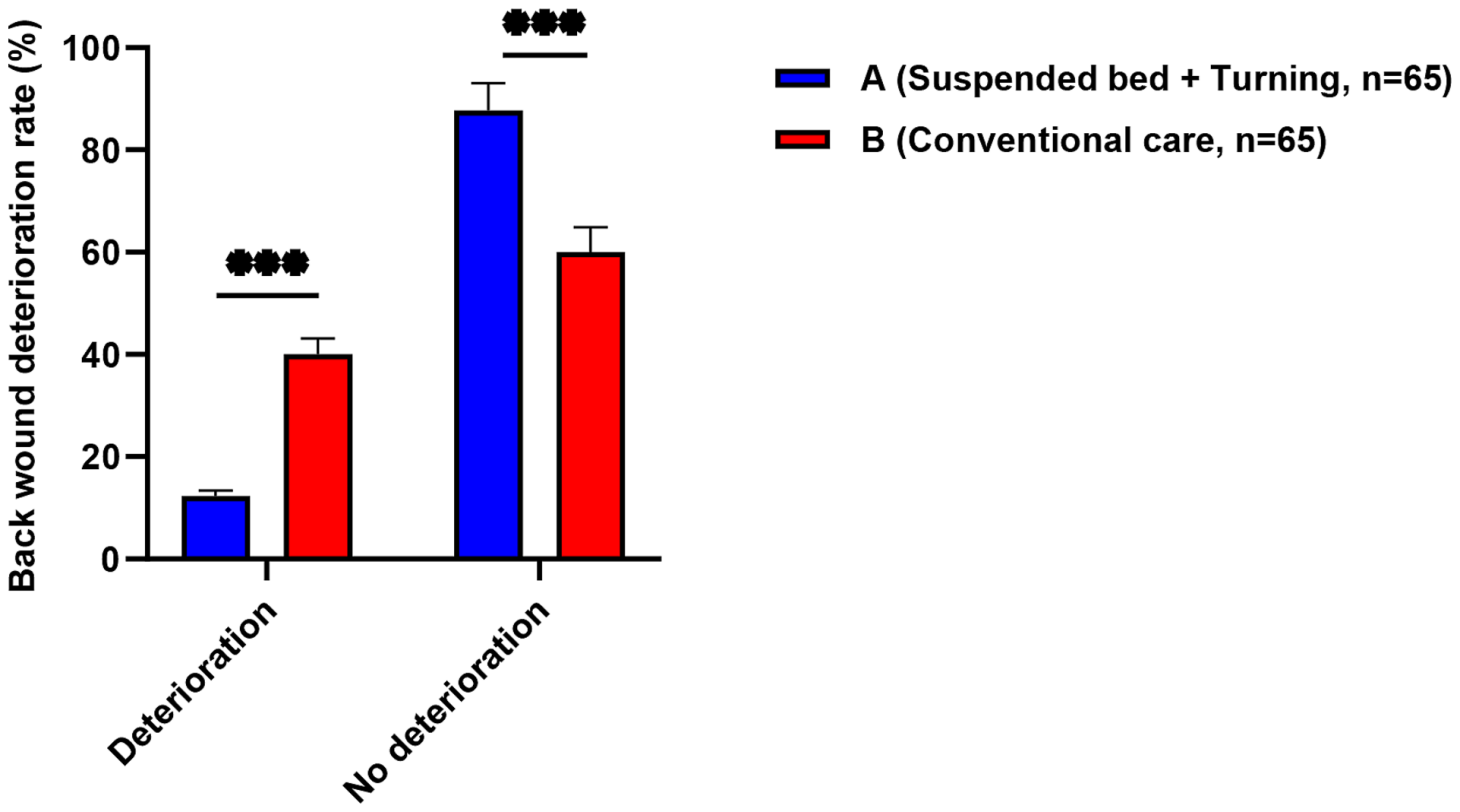
Cumulative risk curves for ventilator-associated pneumonia (VAP).

Postoperative dynamic monitoring showed that airway secretion scores on postoperative day 3 and day 7 were better in Group A than in Group B [(1.75 ± 0.39) points vs. (2.47 ± 0.47) points, *p* < 0.001; (1.51 ± 0.30) points vs. (2.15 ± 0.38) points, *p* < 0.001; Figure 3], indicating a clear advantage of the wound-traversing incision strategy in promoting airway secretion drainage and maintaining airway humidification. Regarding infection outcomes, the median time to VAP occurrence was later in Group A than in Group B [(13.67 ± 1.50) days vs. (8.77 ± 1.68) days, *p* < 0.001]. Although the VAP incidence was numerically lower in Group A (22.0% vs. 40.0%), the difference did not reach statistical significance (*p* = 0.054). Cumulative risk curve analysis (Figure 4) showed that the two curves separated early, and the risk in Group A remained consistently lower over time, suggesting a potential trend for wound-traversing incision to delay and reduce VAP risk.

### Association of systematic postural management with wound protection

**Figure 5:**
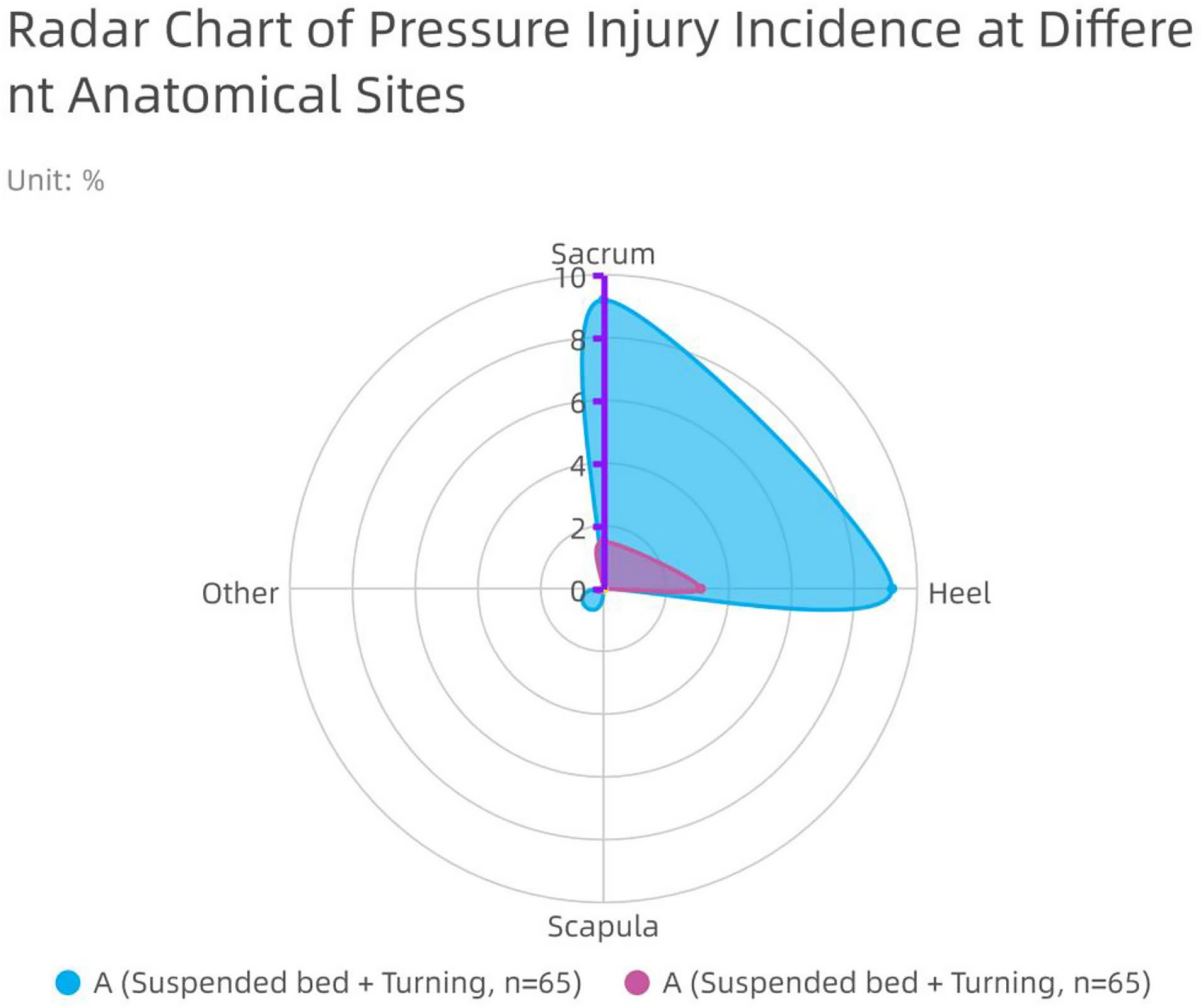
Comparison of back wound deterioration between group.

**Figure 6:**
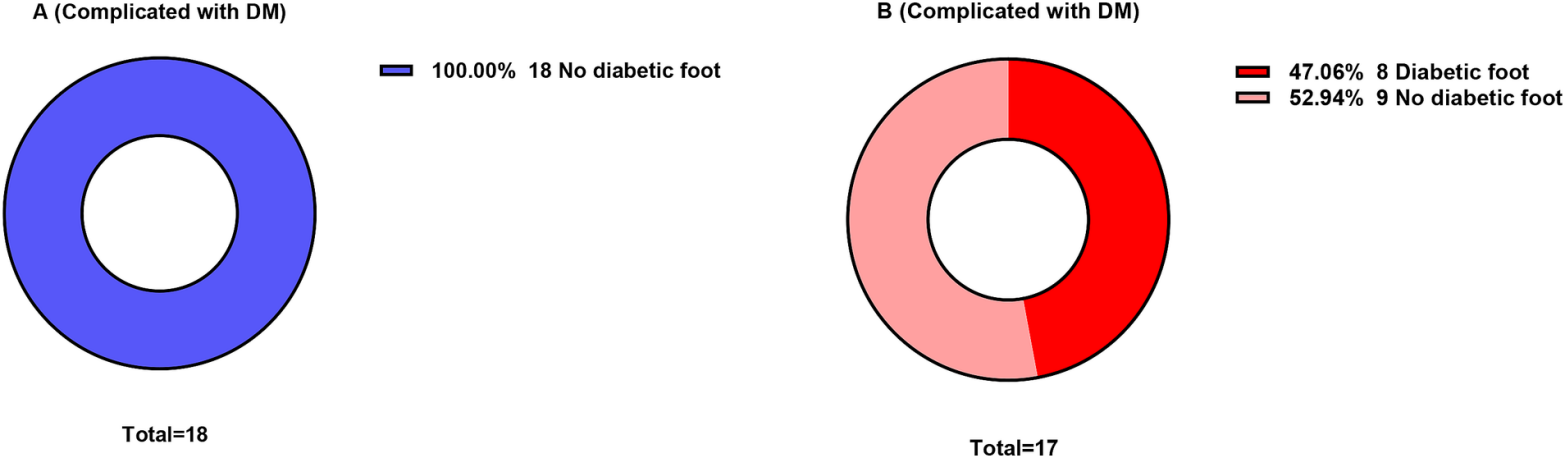
Radar chart of pressure injury incidence at different anatomical sites in the two groups.

All patients in Group A received systematic postural management involving air-fluidized beds combined with scheduled turning. The results showed that this dynamic postural management strategy was associated with better wound protection outcomes. The proportion of patients with progressive deepening of back wounds (from partial-thickness to full-thickness) was far lower in Group A than in Group B (12.3% vs. 40.0%, *p* < 0.001) (Figure 5). Concurrently, the incidence of Stage II or higher pressure injuries was also lower in Group A (4.6% vs. 18.5%, *p* = 0.013). Radar chart analysis (Figure 6) showed that Group A performed better in protecting multiple pressure points, including the sacrococcygeal area and heels.

### Associations of intensified preventive care with diabetic foot outcomes

**Figure 7:**
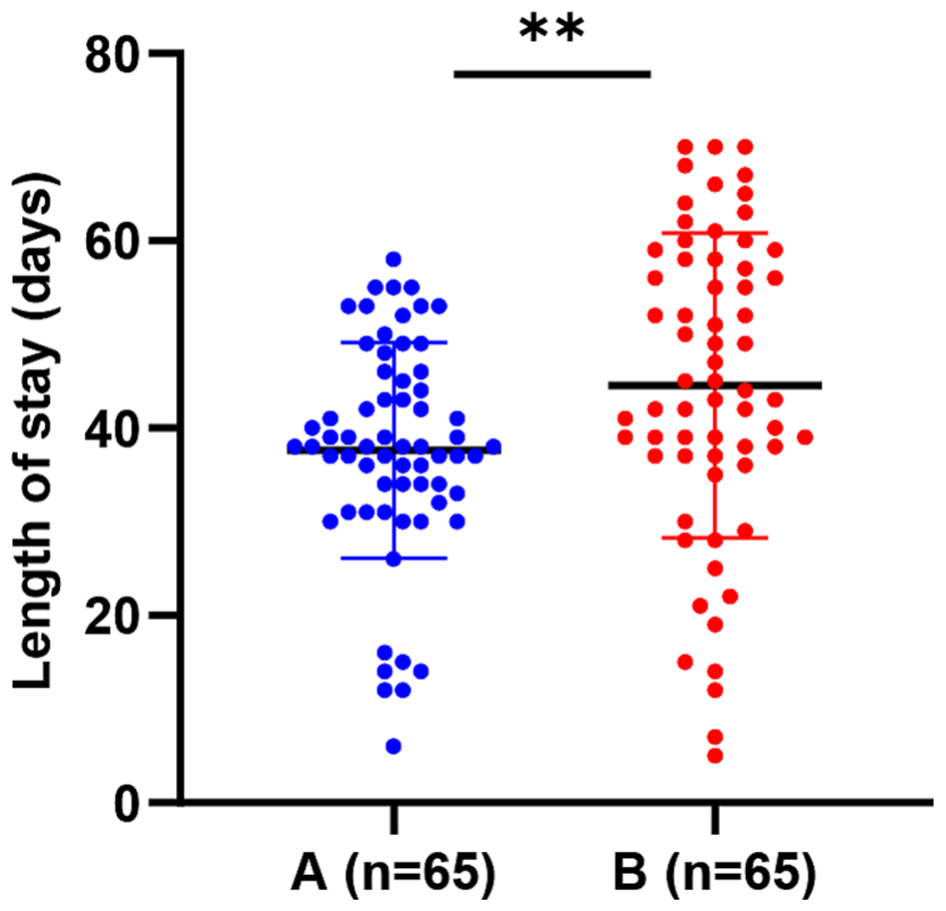
Comparison of diabetic foot incidence in the diabetic patient subgroup.

Within the subgroup of 35 patients with comorbid diabetes (Group A: 18, Group B: 17), intensified preventive care was associated with a markedly lower incidence of diabetic foot compared with conventional monitoring strategies. Patients in Group A initiated an intensified prevention protocol upon admission, including systematic foot risk assessment, standardized offloading interventions, and structured health education. Patients in Group B primarily received conventional blood glucose monitoring without systematically implementing the aforementioned specialized foot care. The results showed that among the 18 patients receiving intensified prevention, not a single case of Wagner grade 1 or higher diabetic foot occurred during hospitalization or within the 3-month follow-up period after discharge (incidence 0%). In contrast, among the 17 patients in Group B, 8 cases (47.1%) developed diabetic foot (Figure 7). The intergroup difference was highly statistically significant (*p* < 0.001).

### Associations of comprehensive care strategy with overall patient prognosis

**Figure 8:**
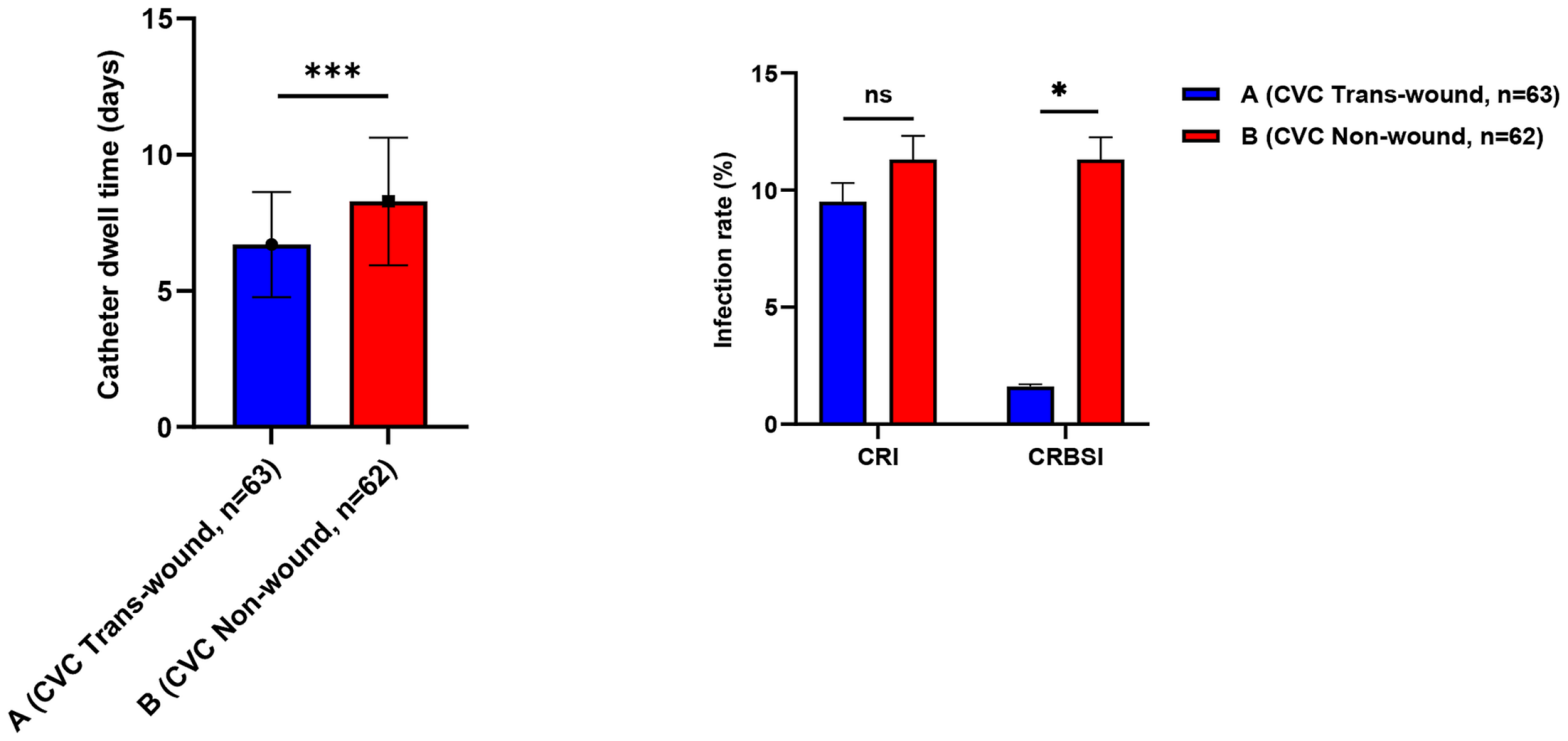
Box plot of total hospital length of stay distribution in the two groups.

The integrated application of diversified nursing strategies was associated with better overall patient outcome indicators in this retrospective cohort. Regarding hospitalization duration, the mean total hospital stay was shorter in Group A than in Group B [(37.66 ± 11.53) days vs. (44.55 ± 16.26) days, *p* = 0.006]. When stratified by survival status, among survivors, the mean LOS was 38.41 ± 10.87 days in Group A (*n* = 60) and 46.35 ± 15.16 days in Group B (*n* = 51); among non-survivors, the mean LOS was 22.60 ± 7.47 days in Group A (*n* = 5) and 34.14 ± 11.52 days in Group B (*n* = 14). Two patients (3.1%) in Group A and four patients (6.2%) in Group B were discharged against medical advice. Regarding severe complications, the incidence of MODS was lower in Group A than in Group B (15.4% vs. 36.9%, *p* = 0.005). Finally, the all-cause mortality rate was only 7.7% in Group A, lower than the 21.5% in Group B (*p* < 0.001). Final outcome composition analysis further confirmed that the cure and discharge rate was as high as 89.2% in Group A, compared to 58.5% in Group B (*p* < 0.001; Table 2). Multivariate Cox regression analysis indicated that receiving the comprehensive care strategy remained an independent protective factor for reducing patient mortality risk after adjusting for potential confounding factors such as age and burn area. As shown in Table 3, the comprehensive care strategy remained associated with reduced mortality risk (HR = 0.32, 95% CI: 0.12–0.85, *p* = 0.021) after adjustment for confounders. Severe inhalation injury (compared with mild) was independently associated with increased mortality risk (HR = 3.45, 95% CI: 1.28–9.32, *p* = 0.014) (Figure 8).

**Table 2:** Comparison of comprehensive clinical outcomes between groups.

Outcome indicator	Group A (*n* = 65)	Group B (*n* = 65)	Statistical test	*p*-value
MODS incidence, *n* (%)	10 (15.4)	24 (36.9)	*χ*^2^ = 7.806	0.005
Mortality, *n* (%)	5 (7.7)	14 (21.5)	*χ*^2^ = 5.200	0.023
Cure and discharge rate, *n* (%)	58 (89.2)	38 (58.5)	*χ*^2^ = 16.496	<0.001

MODS, multiple organ dysfunction syndrome.

**Table 3:** Multivariate Cox regression analysis for in-hospital mortality.

Variable	HR	95% CI	*p* value
Comprehensive care strategy (Group A vs. Group B)	0.32	0.12–0.85	0.021
Age (per 10-year increase)	1.15	0.92–1.44	0.215
TBSA (per 10% increase)	1.28	0.98–1.67	0.069
Inhalation injury grade			
Mild	Reference		
Moderate	1.86	0.71–4.87	0.207
Severe	3.45	1.28–9.32	0.014

HR, hazard ratio; CI, confidence interval; TBSA, total body surface area burned. Variables were entered simultaneously into the Cox proportional hazards model.

## Discussion

This study conducted a retrospective cohort analysis to systematically investigate the efficacy of bundled nursing strategies in patients with severe burns combined with inhalation injury. The results demonstrate that, compared with conventional nursing following traditional pathways, a diversified nursing protocol integrating ultrasound-guided intravenous catheterization through burn wounds, tracheostomy via burn wounds, systematic air-fluidized bed posture management, and reinforced diabetic foot prevention was associated with improved multiple key clinical outcomes (23). This was also associated with shorter hospital stays, lower incidence rates of MODS and mortality, and higher rates of cure-upon-discharge (24). However, these findings should be interpreted cautiously, because differences in baseline respiratory severity and ventilator dependence may also have contributed to part of the observed outcome differences.

First, concerning the controversy over invasive procedures on burn wounds, this study offers strong supportive data (25). The conventional view holds that punctures or incisions should be strictly avoided in contaminated or damaged skin areas (26). However, the present results show that under real-time ultrasound guidance, central venous catheterization through burn wounds achieved a reduced CRBSI rate of 1.6%, far lower than the 11.3% observed in the non-wound catheterization group. A shorter indwelling duration should not be interpreted as a beneficial outcome in isolation; rather, in the present context it may reflect that wound-traversing ultrasound-guided access provided a feasible and efficient route for timely catheter use and removal or replacement when clinically appropriate, while the more important safety finding was that this strategy was also associated with a lower CRBSI rate. This seemingly counter-intuitive outcome may be attributed to several key factors: ultrasound guidance enables visualization, avoiding vascular injury from blind puncture; the trans-wound pathway is often shorter and more direct, reducing subcutaneous catheter tunneling; and combined with standardized maintenance, these factors jointly establish a safety barrier. This seemingly counter-intuitive outcome may be attributed to several key factors: ultrasound guidance enables visualization, avoiding vascular injury from blind puncture (27); the trans-wound pathway is often shorter and more direct, reducing subcutaneous catheter tunneling (28); and combined with standardized maintenance, these factors jointly establish a safety barrier (29). This finding suggests that, under strict technical safeguards, prudent evaluation and selective breakthrough of traditional taboos may offer a feasible alternative for establishing vascular access in critically ill patients (30). Similarly, tracheostomy performed through neck burn wounds demonstrated clear advantages in improving airway secretion scores and delaying the median time to VAP onset by nearly 5 days. Although the reduction in VAP incidence did not reach statistical significance (22.0% vs. 40.0%, *p* = 0.054), the trend toward delayed onset and the improvement in secretion clearance are consistent, indicating that this strategy—by integrating wound drainage with airway management—may offer a beneficial option for reducing early pulmonary infection risk (31). However, this airway-related finding should be interpreted cautiously. Because the comparison group included patients managed with orotracheal/nasotracheal intubation as well as tracheostomy through intact skin, the observed differences in airway secretion clearance and VAP timing may not be attributable solely to the wound-traversing tracheostomy approach itself. Part of the observed advantage may also reflect the known clinical differences between tracheostomy and translaryngeal intubation, particularly with respect to airway toilet, secretion management, and pulmonary infection risk.

Second, this study found that dynamic posture management plays a crucial role in preventing progressive deepening of burn wounds (32). The data suggest that patients receiving air-fluidized bed combined with planned turning management had significantly lower rates of back wound deepening (12.3% vs. 40.0%) and the incidence of stage II or higher pressure injuries (4.6% vs. 18.5%). This goes beyond mere pressure injury prevention; its core value lies in actively protecting residual epithelial tissue (33). For patients with extensive burns, preventing wound deepening equates to preserving precious autologous skin sources to the greatest extent, directly influencing repair outcomes and functional prognosis (34). Therefore, elevating dynamic posture management to the strategic level of “wound-protective therapy” should become a central tenet in critical burn nursing (35).

Third, this study integrated diabetic foot prevention into acute-phase burn care and confirmed its outstanding effectiveness. In the diabetic subgroup, the reinforced prevention group achieved a breakthrough outcome of zero diabetic foot occurrence (0% vs. 47.1%). This stark contrast indicates that during the burn stress period, the pathological progression of diabetic foot is dramatically accelerated, and routine blood glucose monitoring is insufficient for protection. Systematic foot assessment, pressure-relief interventions, and health education constitute an effective early barrier. This reflects the modern nursing concept of extending from “disease treatment” to “health management”—namely, that nursing activities should prospectively block long-term complication chains that severely affect quality of life, achieving truly comprehensive care.

This study has several limitations. First, this was a retrospective single-center study, which is inherently subject to selection bias and residual confounding. Although no statistically significant differences were observed in most baseline variables, Group A included a numerically higher proportion of patients with mild inhalation injury, and some patients may also have had less dependence on mechanical ventilation. These factors may reflect lower baseline respiratory severity, greater mobility, and reduced sedation exposure, which could themselves contribute to better clinical outcomes. Therefore, some of the differences observed in complications and prognosis may not be attributable solely to the nursing care bundle. Another important limitation is that the airway comparison was not fully homogeneous. In Group B, the conventional airway pathway included both orotracheal/nasotracheal intubation and tracheostomy through intact skin, whereas the airway analysis in Group A focused on patients undergoing wound-traversing tracheostomy. Therefore, the observed differences in secretion clearance and VAP-related outcomes may have been influenced not only by the tracheostomy route (through burn wound vs. conventional route), but also by the airway modality itself (tracheostomy vs. translaryngeal intubation). As a result, this comparison should not be interpreted as a pure assessment of wound-traversing tracheostomy alone. Second, because of the retrospective design, we were unable to fully adjust for potentially important clinical factors such as duration of mechanical ventilation, depth of sedation, and mobility status during hospitalization. Third, the study sample was derived from a single institution, which may limit the generalizability of the findings. Future prospective, multicenter studies with more detailed adjustment or stratification for inhalation injury severity, ventilator dependence, sedation exposure, and mobility status are needed to further clarify the independent effect of the nursing care bundle.

## Conclusion

In summary, in patients with severe burns combined with inhalation injury, the implementation of a diversified bundled nursing strategy integrating prudent trans-wound invasive procedures, scientific dynamic posture management, and proactive metabolic complication prevention was associated with favorable clinical outcomes and appeared to be safe in this cohort. The findings suggest that this strategy may help reduce the risk of complications such as catheter-related bloodstream infection, progressive wound deepening, and diabetic foot, may delay the onset of ventilator-associated pneumonia, and may be associated with shorter hospital stay, lower incidence of multiple organ dysfunction syndrome, reduced mortality, and improved recovery. However, as this was a retrospective study and some baseline differences in respiratory severity and ventilator dependence may have influenced the outcomes, the observed benefits should be interpreted cautiously. Overall, this study provides additional clinical support for the potential value of comprehensive, precisely targeted nursing interventions in critical burn care and may help inform future refinement of burn nursing practice.

## Data Availability

The original contributions presented in the study are included in the article/Supplementary material, further inquiries can be directed to the corresponding author.
