# Supplementary figures and images for: Associations of a nursing care bundle with clinical outcomes in patients with severe burns and inhalation injury: a retrospective cohort study
Source: Front Med (Lausanne). 2026 May 7;13:1809662. doi: 10.3389/fmed.2026.1809662 (PMC13190189; doi:10.3389/fmed.2026.1809662)

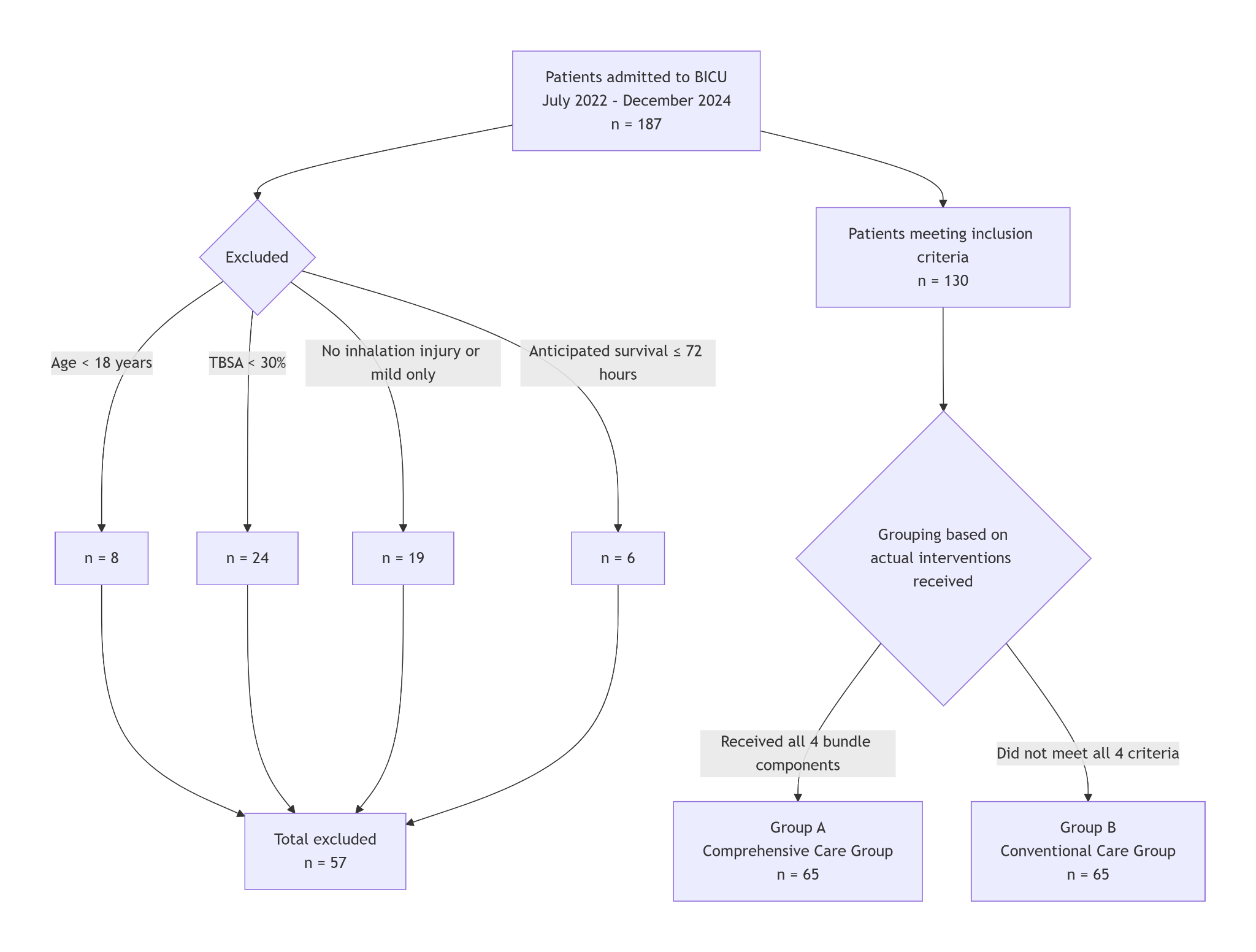

Supplement: Supplementary file 1 [file Image_1.TIFF]
